# Prevalence of *Clostridium botulinum* in Retail Peanut Butters from a 2007 Survey in Ottawa, Canada

**DOI:** 10.1007/s00284-024-03843-1

**Published:** 2024-08-23

**Authors:** Richard A. Harris, Jeff Bussey, Annika Flint, Madeleine Blondin-Brosseau, Kelly Weedmark, John W. Austin

**Affiliations:** Bureau of Microbial Hazards, Health Canada, Ottawa, ON Canada

## Abstract

The spore-forming, anaerobic bacterium, *Clostridium botulinum*, can cause intestinal toxemia (colonization) botulism in adults and infants by colonizing the gut and producing botulinum neurotoxin in situ. In 2006, peanut butter was identified as a lab-confirmed source of *C. botulinum* spores for an adult colonization botulism case in Canada. It is recommended for infants to be exposed to peanut butter at an early age to help prevent the development of a peanut allergy, yet the prevalence of *C. botulinum* in retail peanut butters is currently unknown. This report details a survey that was conducted in 2007 for the presence of viable *C. botulinum* spores in 92 peanut butters and 12 other nut butter spreads obtained from retail grocery stores in Ottawa, Canada. Samples were tested for viable *C. botulinum* spores by detecting botulinum neurotoxin in enrichment cultures by mouse bioassay. Three of the peanut butters from the entire survey of nut butter spreads (3/104, 3%) produced cultures containing botulinum neurotoxin. Whole genome sequencing performed on one isolate from this survey, as well as a clinical isolate and peanut butter isolates associated with the 2006 adult colonization case revealed that all *C. botulinum* isolates contained a full-length chromosomal *bont/A1* gene within an ha–orf + cassette. This study identifies retail peanut butters as a potential source of viable *C. botulinum* spores at the time of sampling. Whether peanut butter represents a food category that may be contributing to the incidence of infant botulism has yet to be determined.

## Introduction

*Clostridium botulinum* is a Gram-positive, spore-forming, anaerobic bacterium that is ubiquitous in nature worldwide [1]. *C. botulinum* and other neurotoxic clostridia, including some strains of *C. baratii* and *C. butyricum*, produce botulinum neurotoxin (BoNT) that causes botulism by cleaving the presynaptic vesicle proteins responsible for acetylcholine release into neuromuscular junctions, resulting in flaccid paralysis [2]. Botulism is classified according to the route of exposure to BoNTs. Foodborne botulism is a result of ingesting foods containing BoNT that were previously contaminated with viable *C. botulinum* and permitted to grow, while adult intestinal toxemia botulism and infant botulism are caused by colonization of the intestines by *C. botulinum* with subsequent production of BoNTs in situ. Infant botulism is most common in children approximately three to four months old, which coincides with the typical age of introducing solid foods [3]. This represents a potential new source for ingesting spores and is also associated with a disruption to gut bacterial taxa that could be associated with susceptibility to colonization [4]. Healthy adults are normally resistant to *C. botulinum* colonization unless the gut microbiota is disturbed by gastrointestinal abnormalities including previous bowel surgery, Crohn’s disease, Meckel’s diverticulum, or recent use of antibiotics [5]. Adult and infant colonization botulism are primarily associated with group I (proteolytic) *C. botulinum* types A and B, although some cases of infant botulism have been caused by neurotoxic strains of *C. baratii* type F [6] and *C. butyricum* type E [7], as well as one instance of group II *C. botulinum* type E [8].

Infant botulism is now the most common form of botulism in Canada with approximately 4.3 cases per million live births annually [9]. The ubiquity of *C. botulinum* spores in the environment and the sporadic nature of infant botulism make source attribution a significant challenge. Previous cases of infant botulism have implicated the ingestion of contaminated honey, as well as outdoor soil and indoor dust as primary sources of exposure to spores [10], yet the origin of infection remains unknown for the majority of cases. From 1979 to 2019, only 5% of all infant botulism cases in Canada were attributed to a laboratory-confirmed source of *C. botulinum* spores, all of which were caused by ingestion of honey [9]. It is now well known not to feed honey to infants less than one year of age, yet other foodborne sources of spores are likely unrecognized. In 2006, an isolate of group I *C. botulinum* type A from the enema fluid of an adult colonization botulism case in Canada was genetically matched to an isolate of *C. botulinum* from a consumed retail peanut butter [11]. In another case of adult colonization botulism in Canada, group I *C. botulinum* type A was isolated from an opened retail peanut butter in the patient’s home, but did not match the serotype of an isolate recovered from the patient’s stool [11].

Fruits, legumes, mushrooms, and vegetables can be contaminated during harvesting or processing by *C. botulinum* spores that are ubiquitous in terrestrial soils [1]. According to published scientific literature, the process of making peanut butter involves dry or oil roasting the shelled peanuts at temperatures and exposure times that may not be sufficient to destroy all heat-resistant group I *C. botulinum* spores most commonly associated with infant botulism [12]. Since 2013, the Canadian Pediatric Society recommends that allergenic “trigger” foods, including peanuts, be introduced to infants as early as four to six months of age to help prevent the development of severe food allergies [13]. Therefore, peanut butter represents a food category that may be contributing to the incidence of infant botulism and has yet to be fully recognized. This report details a survey performed in 2007 of 92 peanut butters and 12 other nut butter spreads obtained from retailers in Ottawa, Canada, for the presence of viable *C. botulinum* spores. Three containers of peanut butter from this survey (3%) were identified as containing viable group I *C. botulinum* spores. Whole genome sequencing was performed in 2021 on one isolate from this survey, as well as isolates obtained from opened and unopened retail peanut butters and an enema fluid associated with the 2006 case of laboratory-confirmed adult colonization botulism.

## Materials and Methods

### Sampling of Peanut Butters and Other Nut Butter Spreads

In 2007, 48 jars of various brands and types of peanut, almond, pea, and hazelnut spreads were purchased from retail grocery store A in Ottawa. Additional 40 products were purchased from retail grocery store B and 16 products from retail grocery store C. One opened peanut butter associated with the 2006 adult colonization botulism case was obtained from the patient’s home in Ontario and three sealed peanut butters of the same brand as this case but different lot numbers were obtained in 2007 from retail grocery store D in Ontario. The same lot numbers were not available at that time. The jars were stored at room temperature until tested.

### Testing of Enrichment Cultures for Botulinum Neurotoxin by Mouse Bioassay

50 g of peanut butter was taken from the top and bottom of each jar and transferred aseptically to a 250 mL bottle containing 150 mL of deaerated cooked meat medium (Oxoid). The duplicate samples were then vortexed briefly before heat shocking at 60 °C for 20 min. The bottles were then transferred to a shaking incubator at room temperature to cool for 10 min before incubating at 35 °C in an anaerobic chamber for 24 h to four days. Bottles were then left at room temperature in the anaerobic chamber for an additional six days. The cultures were centrifuged at 18,500 × g for 30 min, and 10 mL of supernatant was filtered (0.45 μm) and transferred to 15 mL test tubes. In some instances, the duplicate cultures were combined into one sample for testing. The mouse bioassay was used to determine toxicity as described in MFHPB-16 [14]. All animal use followed protocols approved by institutional (Health Canada) animal care and use committees.

### Most Probable Number (MPN) Determination of *C. botulinum* in Peanut Butter

Three portions of peanut butter at each 50 g, 5 g, and 0.5 g were aseptically transferred from the container to 100 mL of deaerated TPGY media (5% tryptone (BD), 0.5% peptone (Oxoid), 0.4% glucose (BD), 2% yeast extract (BD), and 0.1% sodium thioglycolate (Sigma-Aldrich)) and incubated at 35 °C in an anaerobic chamber for 5 days. A mouse bioassay was performed in duplicate as described above to determine toxicity (indicating a positive culture) and the MPN was calculated as previously described [15].

### Freezing and Thawing of *C. botulinum* Isolates

Cooked meat medium cultures of peanut butter identified as toxic were streaked onto MT-EYE agar plates (McClung Toabe agar (HiMedia) mixed with egg yolk emulsion prepared fresh from homogenizing two egg yolks into 100 mL of sterile saline per 1 L media) and incubated at 35 °C in an anaerobic chamber for 24 h then at room temperature for an additional five days. Single lipase-positive colonies of PB84A (peanut butter from brand D), EF0611A (enema fluid from 2006 adult colonization botulism case), PB0612A (case-associated opened brand D peanut butter), as well as PB10507A, PB20607A, and PB30607A (case-associated brand D peanut butters) were re-streaked onto MT-EYE agar plates and incubated at 35 °C in an anaerobic chamber for 24 h then at room temperature for additional five days. Single colonies were frozen at −80 °C using Microbank cryovials (Pro-Lab Diagnostics) according to the manufacturer without further subculture. In 2021, frozen stocks were streaked onto MT-EYE agar and incubated in an anaerobic chamber at 35 °C for 24 h, then a single colony was inoculated into 25 mL of TPGY and cells were collected by centrifugation for 20 min at 12,000 × g after 24 h of anaerobic growth at 35 °C. Two of the toxic cultures from peanut butters (brand D, crunchy, and brand L, smooth 2) were improperly stored and could not be recovered for sequencing.

### DNA Extraction and Sequencing

Cell pellets were resuspended in 1 mL of gelatin phosphate buffer and 1 mL of 2 × Zymo DNA/RNA Shield (Cedarlane) and held at 4 °C. DNA extractions were performed using the Zymo Quick-DNA HMW MagBead kit (Cedarlane) according to the manufacturer’s protocols (RNAse A treatment without enzymatic lysis). *C. botulinum* libraries were constructed using the NexteraXT DNA Library Preparation Kit and Nextera DNA UD Indexes according to the manufacturer’s instructions (Illumina Inc.). Paired-end Illumina sequencing was performed on a MiSeq instrument (v3 chemistry, 2 × 300 bp) or NextSeq 500 instrument (v2 high output chemistry, 2 × 150 bp). All sequencing were performed according to the manufacturer’s instructions (Illumina Inc.).

### Read Processing, de Novo Genome Assembly, and Genome Annotation

Raw Illumina reads were processed using FastP (v0.20.1) to remove adapter and barcode sequences, correct mismatched bases in overlaps, and filter low-quality reads (Q < 20) [16]. De novo assemblies were generated using Unicycler (v 0.5.0) in normal mode [17]. Error correction was performed using Polypolish v0.4.3 [18] and Polca v4.0.5 (github.com/alekseyzimin/masurca). Assemblies were annotated with PGAP v2023-10-03.build7061 (best-placed reference protein set, GeneMarkS-2+) (github.com/ncbi/pgap). Strains were analyzed with QUAST v 5.0.2 (github.com/ablab/quast).

### Genome Comparisons and BoNT Typing

Average nucleotide identity (ANI) between all *C. botulinum* genomes was calculated using FastANI v1.33 [19]. MUSCLE using MEGA (v11.0.13) [20] was used to align BoNT amino acid sequences and cassettes using a subset of references from BoNTbase (bontbase.org) (github.com/bmh-genomics/bont_aa-typing, f9caf33) and from Uniprot (uniprot.org) (github.com/bmh-genomics/orfx-ha_cluster_DB, 6e13cb2). Maximum likelihood phylogenetic trees based on the Jones–Taylor–Thornton model were constructed and visualized with MEGA (v11.0.13, [20]) using the aligned sequences and 1000 bootstrap replicates. Single-nucleotide variant (SNV) analysis was performed using Breseq (v0.38.1, [21]) using the mutation prediction pipeline and default parameters. Variants were identified using Illumina reads for each isolate and the PB84A, EF0611A3, or PB0612A annotated genomes as references. SNVs were reported for nucleotide positions with at least 20 × coverage*.*

## Results

### Survey of Retail Peanut Butters and Other Nut Butter Spreads in Ottawa, Canada in 2007

A total of 92 peanut butters and 12 other nut butter spreads from 12 different brands and 41 different formulations were tested. Viable *C. botulinum* spores were detected in three of the peanut butters from the survey (3/104, 3%) by detecting the presence of BoNT in filtered supernatants from enrichment cultures with the mouse bioassay (Table 1). A group I *C. botulinum* type A was found in each of the brand D crunchy and smooth peanut butters, and one group I *C. botulinum* type AB was found in brand L smooth peanut butter.

**Table 1:** Detection of viable *C. botulinum* spores in 2007 retail peanut butters and other nut butter spreads by testing for botulinum neurotoxin in enrichment cultures by mouse bioassay

Brand	Spread Descriptions	Containers size (kg)	Containers tested (*n*)	*C. botulinum* detected (*n*)
A	Peanut, crunchy; Peanut, smooth; Peanut, smooth, low fat	0.5, 1, 2	10	0
B	Nut free, crunchy; Nut free, smooth	0.5	2	0
C	Peanut, smooth; Peanut crunchy	1	4	0
D	Peanut, natural, smooth; Peanut, crunchy, Peanut, smooth; Peanut smooth, low fat; Peanut unsweetened, unsalted	0.5, 0.75, 1, 2	28	2 (BoNT/A)
E	Peanut, crunchy; Peanut smooth; Peanut, crunchy, with salt; Peanut, smooth, with salt	0.5	4	0
F	Peanut, crunchy; Peanut, smooth	0.5, 1, 2	12	0
G	Pea; Pea, no salt	0.51	2	0
H	Hazelnut	0.75	2	0
I	Hazelnut, chocolate; Almond; Peanut, crunchy; Peanut, smooth; Peanut, smooth, organic	0.5, 0.75, 1	12	0
J	Peanut, crunchy; Peanut, crunchy, no fat; Peanut, smooth; Peanut, smooth, light	0.75, 1	12	0
K	Peanut, smooth; Peanut, smooth, organic; Peanut, crunchy	0.5	6	0
L	Peanut, smooth; Peanut, natural, smooth; Peanut, smooth, organic; Peanut, natural, crunchy; Peanut, natural, crunchy, organic	0.5	10	1 (BoNT/AB)
Total			104	3 (3%)

### Whole Genome Sequencing of Peanut Butter Isolates

*C. botulinum* genome assembly and annotation metrics for one of the isolates from the peanut butter survey, PB84A (brand D, smooth), as well as an isolate from enema fluid and isolates from opened and unopened brand D peanut butters associated with the 2006 adult colonization botulism case are shown in Table 2. All *C. botulinum* isolates contained a full-length chromosomal *bont/A1* gene within an ha–orf + cassette and a truncated, silent *bont/B* gene. The isolates showed high genomic similarity (ANI scores of 99.97% or higher) and were 96.77–96.85% similar by ANI to *C. botulinum* BoNT/A1 silent B strain CDC_96096 (GenBank accession: CP013857.1). When compared to the peanut butter survey isolate PB84A, SNVs were identified in a nucleoside recognition domain-containing protein (I188I, ATC → ATT) in PB0612A and a flagellar motor switch protein FliM (T2A, ACA → GCA) in PB30607A. No SNVs were identified in EF0611A, PB10507A, or PB20607A. Furthermore, the EF0611A (enema fluid) and PB0612A (case-associated opened peanut butter) differed by one SNV in a nucleoside recognition domain-containing protein (I188I, ATT → ATC), suggesting they are clonal, which confirms the genetic matching observed by pulsed-field gel electrophoresis of these isolates originally described in 2012 [11]. The brand L peanut butter identified as containing *C. botulinum* type AB could not be sequenced due to an error in the freezing process of this strain.

**Table 2:** Draft genome assembly and annotation metrics of *C. botulinum* isolates from retail peanut butters (brand D) and enema fluid. Genomes (NCBI Bioproject PRJNA1085258) were annotated using PGAP and analyzed using Quast

Metric	PB84A	EF0611A	PB0612A	PB10507A	PB20607A	PB30607A
Isolate Origin	Peanut butter Brand D	Enema fluid	Peanut butter Brand D	Peanut butter Brand D	Peanut butter Brand D	Peanut butter Brand D
Year	2007	2006	2006	2007	2007	2007
Source	Retail Survey	Clinical	Patient’s home	Retail not survey	Retail not survey	Retail not survey
Genome Accession #	JBBAYH000000000	JBBAYG000000000	JBBAYF000000000	JBBAYE000000000	JBBAYD000000000	JBBAYC000000000
# Contigs	51	61	69	57	57	78
Chromosome (bp)	3,793,757	3,886,570	3,879,598	3,892,755	3,881,547	3,877,869
GC content (%)	27.96	27.95	27.99	27.96	27.94	27.98
Annotated features	3602	3678	3680	3689	3694	3675
Genes	3443	3516	3508	3519	3526	3504
rRNAs	2	2	2	2	4	2
tRNAs	51	51	56	58	52	51
ncRNAs	4	4	4	4	4	4
Pseudogenes	102	105	110	106	108	114
CRISPR/Cas system	1	1	1	1	1	1

The concentration of *C. botulinum* spores in the three positive batches of peanut butter from the survey was not determined. The opened peanut butter containing isolate PB0612A associated with the 2006 adult colonization botulism case was determined to contain *C. botulinum* at a spore concentration of 14 MPN/kg. The three unopened peanut butters containing isolates PB10507A, PB20607A, and PB30607A tested in connection to this case were determined to have spore concentrations of 18 MPN/kg, 29 MPN/kg, and 210 MPN/kg, respectively.

## Discussion

The results of this survey identified that retail peanut butter in 2007 contained viable *C. botulinum* spores at a prevalence of approximately 3% in the retail marketplace in Ottawa, Canada. The concentration of *C. botulinum* spores in the peanut butters from this survey (14–210 spores/kg) is considerably less than has been reported in honey (5000–80,000 spores/kg) and chamomile (300–400 spores/kg) [22, 23]. Whole genome sequencing revealed that one isolate from the survey, as well as isolates from peanut butters of the same brand and a clinical isolate associated with the 2006 case of adult colonization botulism contained the *bont/A1* gene within an ha–orf + cassette. The high degree of genomic similarity among isolates from different lots of the same peanut butter brand D suggests a common origin of *C. botulinum* spores, such as the same harvesting region or processing plant. The brand L peanut butter produced toxic cultures of type AB, indicating a distinct *C. botulinum* strain and different source of spores. However, the specific geographical origin of peanuts for these two brands is unknown.

The prevalence of *C. botulinum* in raw harvested peanuts is yet to be determined and may vary by geographic region. Peanut butter is typically made by subjecting shelled peanuts to dry or oil roasting in an oven at temperatures and exposure times that are intended to destroy vegetative cells of spoilage organisms, but may not be sufficient to inactivate heat-resistant group I *C. botulinum* spores [12]. The specifics of the industrial processes by which peanut butter is produced are proprietary, so controls and mitigating practices for *C. botulinum* are unknown. Therefore, it is difficult to know if the presence of *C. botulinum* in the final product was due to the influence of processing conditions or a high source of contamination at harvest. Peanut butter may be contaminated by other ingredients that are known to harbor *C. botulinum*, including natural sugars or honey [24], although none of the implicated products in this report contained these additives. A foodborne botulism outbreak in Taiwan in 1986 was caused by group I *C. botulinum* type A from commercially canned unsalted peanuts in water that resulted in 7 hospitalizations and one death [25]. A follow-up investigation revealed that the company responsible did not have the steam pressurized equipment legally required for thermal processing canned low-acid foods. A total of 104 jars from the implicated batch were recalled and 34 tested positive (33%) for BoNT type A. Peanut butter and other nut butter spreads have water activity levels too low for the growth of *C. botulinum*, and therefore have never been found to contain BoNT or been implicated in foodborne botulism outbreaks.

Peanut butter is a healthy, protein-rich food, containing vitamins, minerals, fiber, and unsaturated fats that provide a substantial nutritional benefit to infants and adults alike. According to the Canada Food Guide, peanut butter can be fed to infants as young as 6 months of age by spreading thinly on a cracker or toast to avoid the formation of an airway seal that is difficult to dislodge and may lead to asphyxiation [26]. The Canadian Pediatric Society now recommends the introduction of allergenic “trigger” foods, including peanuts specifically, as early as 4 months of age to infants who are deemed high risk for severe eczema or have a first-degree relative with an associated food allergy [13]. Infants with mild eczema or no family history of food allergies can introduce trigger foods when deemed appropriate by family preferences or cultural practices as early as 6 months of age. This is in alignment with recommendations from other international societies of clinical immunology and allergology [27–29]. The benefits of preventing potentially fatal peanut allergies in children should be considered against the risk of developing infant botulism, which is rare and can be effectively treated with BabyBig® antitoxin when administered early in disease progression. The age distribution of infant botulism globally centers around three to four months old [3]. This coincides with the introduction of solid foods that is associated with a disruption to the gut bacterial taxa [4]. It is currently unknown if changes to the gut microbiota cause susceptibility to *C. botulinum* colonization, or if infants at this age simply ingest more spores from food or the environment. Peanut butter has never been reported as a confirmed association with infant botulism, so whether peanut butter represents a food category that may be contributing to the incidence of infant botulism remains to be fully investigated.

The source of *C. botulinum* spore ingestion is still unknown for the majority of infant botulism cases. Honey is the only long-standing risk factor for infant botulism, and it is now commonly known not to feed honey to infants less than 1 year of age. Previous laboratory-confirmed associations between isolates from infant stool and isolates from the environment indicate that household dust and outdoor soil are also likely sources of exposure [10]. However, it is often challenging for public health officials to follow-up on each case of infant botulism to sample the local environment, and exposure to dust or soil represents a source of spores that cannot easily be avoided in everyday life. Fruits and vegetables are known to harbor *C. botulinum* due their growth in close proximity to the soil and various associations with foodborne outbreaks [1]. Viable spores can be consumed by most healthy adults without the risk of colonization, although cases have been reported for individuals with no history of gastrointestinal problems [11]. Laboratory-confirmed associations for adult colonization cases have implicated commonly consumed foods as sources of spores including, cream of coconut, blackberries, and peanut butter [11, 30]. Despite this knowledge, investigations of botulism outbreaks do not often produce a confirmed source. Only 54% of foodborne botulism cases in Canada were reported as associated with a confirmed food source from 2006 to 2021 [31]. Infant and adult colonization botulism cases are especially difficult to identify sources due to the sporadic nature of these single-case outbreaks. Foodborne botulism outbreaks involving several linked individuals enable epidemiologic identification of commonly ingested foods. The results of this report suggest that the ingestion of peanut butter should be considered as a risk factor for developing colonization botulism in infants and adults. Primary care physicians and public health officials should be made aware of the risk of ingesting peanut butter, along with honey, when conducting food history for these investigations.

## Conclusions

This survey in 2007 identified retail peanut butter as a potential source of viable *C. botulinum* spores. The feeding of peanut butter to infants at a young age is recommended to help prevent the development of severe peanut allergies, and peanut butter has yet to be implicated as a cause of infant botulism. Primary care physicians and public health officials should be made aware of the prevalence of viable *C. botulinum* in peanut butter to inform clinical investigations and aid in the determination of this food as a risk factor for adult and infant colonization botulism.

## Data Availability

Whole genome sequences are available in GenBank under BioProject PRJNA1085258. Raw reads are available at the Sequence Read Archive under the accession numbers SRR28257549–SRR28257554.
